# Social Network Characteristics and Daily Smoking among Young Adults in Sweden

**DOI:** 10.3390/ijerph10126517

**Published:** 2013-11-29

**Authors:** Mikael Rostila, Ylva B. Almquist, Viveca Östberg, Christofer Edling, Jens Rydgren

**Affiliations:** 1 Centre for Health Equity Studies (CHESS), Stockholm University/Karolinska Institutet, Sveaplan, Sveavägen 160, Stockholm SE-106 91, Sweden; E-Mails: yba@chess.su.se (Y.B.A.); vostberg@chess.su.se (V.Ö.); 2 Department of Sociology, Lund University, Box 114, Lund SE-22100, Sweden; E-Mail: christofer.edling@soc.lu.se; 3 Department of Sociology, Stockholm University, Stockholm SE-106 91, Sweden; E-Mail: jens.rydgren@sociology.su.se

**Keywords:** smoking, social networks, homophily, Sweden, young adults, trust

## Abstract

A large number of studies have shown that friends’ smoking behavior is strongly associated with an individual’s own risk for smoking. However, few studies have examined whether other features of social networks, independently or conjointly with friends’ smoking behavior, may influence the risk for smoking. Because it is characterized by the growing importance of friendship networks, the transition from adolescence to young adulthood may constitute a particularly relevant period on which to focus our investigation of network influences on smoking behavior. The aim of this study was therefore to examine the consequences of peer smoking as well as other network characteristics (friends’ other health behaviors, relationship content, and structural aspects of the network) on the risk for smoking among young adults. The data was based on a cross-sectional survey of Swedish 19-year-olds carried out in 2009 (n = 5,695) with a response rate of 51.6%. Logistic regression was the primary method of analysis. The results show that having a large percentage of smokers in one’s network was by far the most important risk factor for daily smoking. The risk of daily smoking was 21.20 (CI 14.24. 31.54) if 76%–100% of the network members smoked. Having a high percentage of physically active friends was inversely associated with daily smoking. The risk of smoking was 0.65 (CI 0.42. 1.00) if 76%–100% of the network members were physically active. No main associations between the other network characteristics (relationship content and structural aspects of the network) and smoking were found. However, there was an interaction between the percentage of smokers in the network and relationship content (*i.e.*, trust, relationship quality and propensity to discuss problems): positive relationship content in combination with peer smoking may increase the risk of smoking. Women with a high percentage of smokers in their networks were also at higher risk of daily smoking than were men with many smoking friends. Hence, it is important to consider the interplay between peer smoking and other network characteristics on the risk of smoking, where features of networks which traditionally are seen as constructive may occasionally provide the impetus to smoke. Future studies should use longitudinal data to study whether these findings reflect peer selection or peer influence.

## 1. Introduction

Smoking is among the leading causes of premature death and of various diseases and health problems in Sweden and in the rest of the world [1]. Adolescence and young adulthood are sensitive periods in terms of taking up smoking, which in turn has long-term consequences for smoking in adulthood. Peer influence and group pressure on smoking are stronger in adolescence and early adulthood than in later adulthood [2]. Few studies have focused on smoking behavior in young adulthood *versus* in adolescence or asked whether smoking behavior is influenced by social network characteristics. In most Western European countries, young adulthood is characterized by great change, including leaving school and perhaps the family home and entering higher education or the labor market [3,4,5]. In Sweden, too, smoking is more common in late adolescence and young adulthood than in any other age group [1]. It is therefore likely that friendships become increasingly important for smoking behavior as young adults grow more and more independent of their parents. A focus on friendship networks may thus enhance the understanding of smoking behavior in early adulthood.

People linked through social ties influence each other’s norms, attitudes, and behaviors [6,7,8]. Social network characteristics can therefore be crucial for starting—and continuing—to smoke. A review of the literature on peer influence on cigarette smoking suggests that the number of friends who smoke is the single most commonly cited peer risk factor for smoking [9]. Associations between the number of friends who smoke and smoking are present across all types of relationships, including friendship dyads, friendship groups, and sets of named friends in both network studies and non-network studies [2,9,10,11,12,13]. Some plausible explanations for an association between peer smoking and the risk for smoking among young persons have been suggested. The decision to smoke is influenced by watching role models who smoke (friends, parents, relatives, *etc*.), assessing the social consequences of smoking, and considering perceived punishments and rewards [9]. Having peers who smoke can lead adolescents and young adults to smoke because they see role models who smoke in their environment, view smoking favorably, and experience fewer punishments and more rewards as a consequence of smoking. However, there is also a possibility that an association between friends’ smoking and smoking behavior reflects the fact that people with similar characteristics tend to interact with each other (homophily or selection). In terms of smoking, homophily means that adolescents and young adults will tend to choose friends who have a similar smoking behavior. There is evidence that both selection and influence play a role in adolescent smoking [10,14,15].

In the context of smoking it is also important to consider network characteristics other than the number of friends who smoke. These may be independently associated with daily smoking or have consequences for the individual’s smoking behavior in combination with peer smoking. Most previous studies show that social relationships with high *relationship content* positively influence health-related behaviors [16]. Social relationships of high quality may prevent adolescents and young adults from adopting maladaptive behaviors such as smoking by causing them to perceive an event as less stressful, or by preventing the adoption of maladaptive coping responses to the stressor [17]. Some studies have, however, suggested that relationships of high quality may increase the likelihood of smoking [18]. Supportive and close friendships may result in more opportunities for influence, leading to similar smoking behaviors among friends [18,19,20,21,22,23]. However, one study found that smoking was positively associated with social support only when peers smoked [24]. In this study we will therefore examine whether relationship content is associated with daily smoking, using measures on overall relationship quality, trust, and the propensity to discuss problems with friends. Moreover, *friends’ health behaviors* other than peer smoking may also influence smoking behavior. It has been argued that social networks may influence health-related norms that, in turn, affect behaviors such as alcohol and cigarette consumption, physical activity, dietary patterns, *etc*. [6,7,16]. People who socialize with one another may also effectively exercise informal social control over the deviant health behaviors of network members [25]. Accordingly, healthier norms and behaviors among network members in general may reduce the likelihood of smoking, while networks dominated by individuals who engage in risky health behaviors may contribute to a higher risk [6,8,26,27,28,29]. In this study we will therefore also examine whether some other health behaviors of friends, such as eating habits and physical activity, influence the risk of smoking. Finally, *structural aspects* of social networks may also influence smoking behavior. One feature of social networks on which effective norms depend is what is sometimes called closure [30]. A closed network facilitates the transmission and maintenance of existing norms among its network members. This is because closed networks provide better opportunities for network members to unite, thereby providing collective sanctions against norm breakers. However, closed networks also facilitate the rapid and effective diffusion of negative as well as positive information, norms, and behaviors as each individual is directly or indirectly linked to the other members of the network [31]. Assuming that some networks include norms that negatively influence smoking, such networks may promote smoking behavior if they are characterized by closure. Furthermore, other structural aspects of the social network may also influence smoking behavior. The influence of peers on health behaviors such as the decision to start or continue to smoke may be stronger when friends live in close proximity and when friends meet more often [8]. For instance, it is possible that such network characteristics lead to an additionally increased risk of daily smoking when friends’ attitudes, norms, and behaviors support smoking. Thus, in this study we will examine whether network closure, frequency of contact with friends and share of friends living same neighborhood influence the risk of daily smoking.

To conclude, the present study will examine the influence of network characteristics on the risk for smoking among Swedish young adults at the age of 19. We are also interested in the interplay between friends’ smoking behavior and other network characteristics on the risk for smoking. The specific research aims of this paper are to: (1) study the distribution of various network characteristics such as health behaviors of friends (*i.e.*, smoking, physical activity and eating habits), relationship content (*i.e.*, relationship quality, trust, propensity to discuss problems with friends), and structural aspects (*i.e.*, frequency of contact, network closure, friends in the neighborhood) among men and women, respectively; (2) examine the association between the aforementioned network characteristics and the risk for daily smoking after adjustment for confounders; and (3) determine whether the association between peer smoking and daily smoking is modulated by any of the network characteristics or sociodemographic factors.

## 2. Methods

### 2.1. Survey Data

We use data from a unique Swedish survey on *Social Capital and Labor Market Integration*, carried out in 2009 [32]. Table 1 shows descriptive statistics of smoking prevalence and control variables included in the study sample. A telephone interview was conducted by Statistics Sweden on a sample of 5,695 19-year-olds. Informed consent was obtained from each interviewee included in the study. The Regional Ethical Review Board of Stockholm (2008/580-31) approved the study because it is based on informed consent from the respondents. The sample was based on three different cohorts of Swedes born in 1990: (a) all individuals with at least one parent born in Iran; (b) 50% of all individuals with at least one parent born in (former) Yugoslavia; and (c) a simple random sample of 2,500 individuals with two Swedish-born parents. These cohorts were selected as they represent some of the largest groups of young adults of foreign background who have been present in the Swedish society over the past several decades. A total of 2,942 interviews were completed, resulting in a response rate of 51.6%. The largest percentage of the non-response, 37.6%, was for *not-at-home*. The refusal rate was 8.1%. The response rate was lower among individuals who had not finished (and were not about to finish) upper secondary school and had lower school grades and less educated parents [33]. We examined various network characteristics and risk for daily smoking by means of logistic regression.

### 2.2. Variables

#### 2.2.1. Friend’s Health Behaviors

Of specific interest for the current study, the questionnaire contained questions about social networks. The respondents (egos) were asked to name the five people with whom they met and socialized most often during their leisure time (alters). The variable *friends smoke* was measured using the question, “Does Alter # smoke?” (yes; no). The variable *friends physically active* was measured using the question, “Does Alter # exercise or play sports (at least half an hour of exercise or sports per occasion)?” (yes; no). The variable *friends eat healthy food* was measured by a question on whether Alter # eats healthy food (yes; no).

**Table 1:** Descriptive statistics of smoking prevalence and control variables included in the study sample, 19-year-old men and women.

	Men		Women		Total	
	N	%	N	%	N	%
**Daily smoking**						
Yes	193	13.1	298	21.1	491	17.0
No	1,276	86.9	1,117	78.9	2,393	83.0
**Parents’ social class**						
Higher non-manuals	209	14.4	228	16.1	437	15.2
Medium non-manuals	380	26.1	372	26.2	752	26.1
Lower non-manuals	102	7.0	108	7.6	210	7.3
Skilled workers	336	23.1	324	22.8	660	22.9
Unskilled workers	321	22.0	257	18.1	578	20.1
Farmers	17	6.3	22	1.5	39	1.4
Self-employed	91	1.2	109	7.7	200	7.0
**School grades**						
Quartile 1	215	14.9	426	30.1	641	22.5
Quartile 2	338	23.5	408	28.8	746	26.1
Quartile 3	401	27.9	297	21.0	698	24.4
Quartile 4	485	33.7	285	20.1	770	27.0
**Civil status**						
Married or boyfriend/girlfriend	450	30.3	543	37.6	993	33.9
No partner	1,037	69.7	903	62.4	1,940	66.1
**Parents’ country of birth**						
Sweden	691	46.3	691	47.7	1,382	47.0
Yugoslavia	478	32.0	450	31.1	928	31.5
Iran	325	21.8	307	21.2	632	21.5
**Ego’s alcohol consumption**						
Once a month or more seldom	866	59.0	934	66.1	1,800	62.5
More than once a month	601	41.0	479	33.9	1,080	37.5
**Ego’s physical activity**						
Yes	1,101	74.9	995	70.2	2,096	72.6
No	368	25.1	423	29.8	791	26.9
**Ego’s eating habits**						
Very important or fairly important	1,028	70.2	1,092	77.1	2,120	73.6
Not important	436	29.8	325	22.9	761	26.4

n = 2,942.

#### 2.2.2. Relationship Content

*Relationship quality* was based on the question, “How good do you think your relationship with Alter # is?” (1–5). The quality measure was calculated as the number of relationships to which the respondent assigned the value 4 or 5 (good or very good). *Trust* was derived from the question, “How much do you trust Alter #?” (1–5). Trust was calculated as the number of relationships to which the respondent assigned the value 4 or 5 (much or very much). The variable on the propensity of *discussing problems* was framed as, “Is this a person (Alter #) with whom you would be able to discuss an important personal problem?” (yes; no).

#### 2.2.3. Structural Aspects

*Frequency of contact* was measured with the question, “How often do you usually meet Alter #?” The values ranged from 1 (daily) to 6 (rarely or never). Frequency of contact was calculated as the number of relationships to which the respondent assigned the value 1 or 2 (daily or several times a week). The variable *friends in the neighborhood* was based on the question, “Does he/she (*i.e.*, Alter #) live in the same neighborhood as you?” (yes; no). Network closure is a widely used concept in sociological research and refers to networks with few links or bridges to adjacent networks [30]. The measurement of *network closure* was derived by asking whether or not alters know each other, and if they do, how well (not so well; fairly well; or very well). Network closure was subsequently calculated as the number of pairs of alters who knew each other very well.

All variables on network characteristics were examined as percentages in the empirical analyses. The variables were then grouped into four categories, 0%–25%, 26%–50%, 51%–75%, and 76%–100%. For the sake of interpretation the variables were dichotomized (e.g., 0%–50% and 51%–100%) in the interaction analysis.

#### 2.2.4. Daily Smoking

*Smoking* is measured with a question, “Do you smoke daily?” (yes; no). Consequently, those who reported that they smoke daily were considered daily smokers.

#### 2.2.5. Control Variables

The analyses were adjusted for gender, parents social class, school grades, civil status, parents’ country of birth, and ego’s health behaviors (for descriptive statistics on control variables, see Table 1). *Parents’ social class* was measured using the Swedish socioeconomic classification (SEI) [34]. This information was divided into seven categories: higher non-manuals; medium non-manuals; lower non-manuals; skilled workers; unskilled workers; farmers; and self-employed. The dominance scale (*i.e.*, choosing the higher-status occupation) was used in cases where parents belonged to different SEI groups. *School grades* in the 9th grade were based on the score of the individual’s sixteen top subjects. The possible grades were: no grade/fail (0 points); pass (10 points); pass with distinction (15 points); and pass with special distinction (20 points). The measure of school grades thus ranged from 0 and 320 and was categorized into quartiles in the empirical analyses. Information about *civil status* was dichotomized into partner (*i.e.*, married or boyfriend/girlfriend) and no partner. Because the data used were based on an ethnically stratified sample, all the analyses were also adjusted for *parents’ country of birth.* Finally, we also adjusted for ego’s health behaviors. *Ego’s alcohol consumption* is measured with the question, “How many times during the past 12 months did you drink alcohol and become intoxicated?” *Ego’s physical activity* is measured with the question, “Do you exercise regularly at least once per week during your spare time? The variable *ego’s eating habits* were measured with a question, “How important is it that the food you eat is healthy?”

### 2.3. Modeling Strategy

The descriptive tables (Table 1 and Table 2) show the distribution of individuals by each variable in total and by gender. Our sample was too small to perform gender-specific analyses in the multivariate analyses (Table 2 and Table 3) and this is also the reason why many variables are dichotomized in the empirical analyses (see Table 4). The strategy in the analyses in Table 3 is to examine the association between various network characteristics and the risk for daily smoking after adjusting for several possible confounders. Model 1 is adjusted for gender and parents’ country of birth. Model 2 examines the contribution of the parents’ social class, school grades, civil status, and ego’s health behaviors, while Model 3 is adjusted for control variables and network characteristics. Since it has been suggested that the number of peers who smoke is the single most important network characteristic for adolescent smoking, Table 4 examines the interaction between peer smoking status and other network characteristics and the risk for daily smoking after adjusting for control variables. Table 4 also examines the interaction between peer smoking and a selection of sociodemographic variables.

## 3. Results

Table 1 shows the distribution of smoking prevalence and control variables included in the study sample. The results suggest that a higher percentage of women in the sample are daily smokers when compared to men. Most respondents have parents with a manual class background (unskilled or skilled worker) or parents who belong to the medium non-manual class. Women have higher school grades when compared to men: a higher percentage of women belong to the quartile with the highest school grades (*i.e.*, Quartile 1). The results also suggest that most respondents are single and born in Sweden. Finally, most men and women in the sample drink alcohol to excess once a month or less, they are physically active, and they believe that good eating habits are important.

Table 2 shows frequencies and the percentage distribution by network characteristics in the sample used in this study. The results suggest that a larger share of women’s peers smoke: compared to men, a higher percentage among women report that 76%–100% of their friends smoke daily. A higher percentage of women also report that their friends are physically inactive when compared to men. On the other hand, women’s networks are characterized by healthier eating habits: about 27% of women report that 76%–100% of their friends eat healthy food while the corresponding number for men is 20%. The results further suggest that most respondents experience their social relationships as being of high quality, *i.e.*, a high share of the respondents report good or very good relationships to 76%–100% of their friends. Here, no profound gender differences in relationship quality are found. Morever, most men and women report high levels of trust toward their closest friends: about 70% of the respondents report that they trust 76%–100% of their friends very much or much. However, the results reveal that women can discuss problems with a larger percentage of their network members compared to men. Concerning the structural aspects of the network, the results suggest that a higher percentage of men report that most of their network members live in the same neighborhood, while a higher percentage of women report that their friends know one another (network closure). Finally, the results suggest that men meet their friends more often than do women: about 66% of all men and 54% of all women report that they meet 76%–100% of their friends at least once a week.

The results in Table 3 suggest strong associations between the health behaviors of friends and the risk for daily smoking among egos. In particular, peer smoking has a very strong association with ego’s risk for daily smoking, even after adjusting for control variables and other network characteristics (Models 2 and 3). The risk of daily smoking is 21.20 (CI 14.24. 31.54) if 76%–100% of the network members smoke in the fully adjusted model (Model 3) when compared to the reference group (0%–25% of friends smoke). This suggests that those with many peers who smoke are at much higher risk for daily smoking than those with few smoking friends.

**Table 2:** Frequencies and percentage distribution by network characteristics, 19-year-old men and women.

	Men		Women		*p*-value (gender diff)	Total	
	N	%	N	%		N	%
FRIENDS’ HEALTH BEHAVIORS							
**Friends smoke (100% = all smoke daily)**					0.050		
0%–25%	833	57.3	775	54.0		1,608	55.7
26%–50%	292	20.1	285	19.9		577	20.0
51%–75%	190	13.1	194	13.4		384	13.3
76%–100%	139	9.6	181	12.5		320	11.0
**Friends physically active (100% = all physically active)**					<0.001		
0%–25%	205	14.1	288	20.1		493	17.0
26%–50%	338	23.2	377	26.3		715	24.8
51%–75%	386	26.5	328	22.9		714	24.7
76%–100%	525	36.1	442	30.8		967	33.5
**Friends eat healthy food (100% = all eat healthy food)**					<0.001		
0%–25%	502	34.5	428	29.8		930	32.2
26%–50%	362	24.9	324	22.6		686	23.7
51%–75%	294	20.2	291	20.3		585	20.2
76%–100%	296	20.4	392	27.3		688	23.8
RELATIONSHIP CONTENT							
**Relationship quality (100% = very good or good relationship to all)**					0.037		
0%–25%	34	2.3	23	1.6		57	2.0
26%–50%	104	7.2	128	8.9		232	8.0
51%–75%	230	15.8	262	18.3		492	17.0
76%–100%	1,086	74.7	1,022	71.2		2,108	73.0
**Trust (100% = trust all very much or much)**					0.583		
0%–25%	38	2.6	31	2.1		69	2.3
26%–50%	137	9.4	119	8.2		256	8.9
51%–75%	245	16.9	252	17.4		497	17.2
76%–100%	1,034	71.1	1,033	71.3		2,067	71.5
**Discuss problems (100% = can discuss problem with all)**					<0.001		
0%–25%	82	5.6	32	2.2		114	3.9
26%–50%	174	12.0	136	9.5		310	10.7
51%–75%	316	21.7	253	17.6		569	19.7
76%–100%	882	60.7	1,014	70.7		1,896	65.6
STRUCTURAL ASPECTS							
**Frequency of contact (100% = meet all at least once a week)**					<0.001		
0%–25%	102	7.0	121	8.4		223	7.7
26%–50%	141	9.7	235	16.4		376	13.0
51%–75%	255	17.5	299	20.8		554	19.2
76%–100%	956	65.7	780	54.4		1,736	60.0
**Network closure**					<0.001		
Not all friends are friends	729	53.2	506	38.4		1,235	45.9
All friends are friends	641	46.8	813	61.6		1,454	54.1
**Friends in the neighborhood (100% = all in same neighborhood)**					0.020		
0%–25%	735	50.6	746	52.0		1,481	51.3
26%–50%	304	20.9	338	23.6		642	22.2
51%–75%	190	13.1	182	12.7		372	12.9
76%–100%	225	15.5	169	11.8		394	13.6
**n**	1,494		1,448			2,942	

**Table 3:** The association between network characteristics and daily smoking among egos, 19-year-old men and women, prevalence ratios (PR).

	Model 1	95% CI/*p*-value		Model 2	95% CI/*p*-value		Model 3	95% CI/*p*-value	
	PR			PR			PR		
FRIENDS’ HEALTH BEHAVIORS									
**Friends smoke**		<0.001			<0.001			<0.001	
0%–25%	1.00			1.00			1.00		
26%–50%	4.94	3.54	6.88	3.91	2.73	5.58	3.92	2.68	5.72
51%–75%	13.94	10.04	19.35	9.78	6.86	13.92	9.98	6.84	14.57
76%–100%	30.25	21.59	42.39	20.45	14.19	29.49	21.20	14.24	31.54
**Friends physically active**		<0.001			<0.001			0.042	
0%–25%	1.00			1.00			1.00		
26%–50%	0.74	0.57	0.97	0.88	0.66	1.19	1.06	0.74	1.51
51%–75%	0.43	0.32	0.58	0.61	0.44	0.84	0.73	0.49	1.08
76%–100%	0.24	0.17	0.32	0.38	0.27	0.53	0.65	0.42	1.00
**Friends eat healthy food**		<0.001			<0.001			0.328	
0%–25%	1.00			1.00			1.00		
26%–50%	0.86	0.67	1.11	1.06	0.80	1.41	1.34	0.95	1.87
51%–75%	0.59	0.44	0.78	0.77	0.56	1.07	1.01	0.69	1.48
76%–100%	0.43	0.32	0.57	0.66	0.48	0.92	1.03	0.69	1.54
RELATIONSHIP CONTENT									
**Relationship quality **			0.052			0.047		0.020	
0%–25%	1.00			1.00			1.00		
26%–50%	0.69	0.29	1.63	0.75	0.28	1.98	0.72	0.20	2.61
51%–75%	1.30	0.59	2.87	1.49	0.60	3.68	1.68	0.47	6.05
76%–100%	1.04	0.48	2.25	1.38	0.57	3.32	1.78	0.50	6.33
**Trust **		0.007			0.100			0.275	
0%–25%	1.00			1.00			1.00		
26%–50%	0.88	0.42	1.86	0.69	0.31	1.54	0.52	0.17	1.58
51%–75%	1.48	0.74	2.95	1.19	0.56	2.51	0.87	0.30	2.56
76%–100%	0.98	0.50	1.91	0.91	0.44	1.86	0.73	0.25	2.14
**Discuss problems **		0.100			0.449			0.206	
0%–25%	1.00			1.00			1.00		
26%–50%	1.62	0.81	3.27	1.48	0.70	3.15	2.65	1.08	6.52
51%–75%	2.12	1.09	4.11	1.72	0.84	3.50	2.19	0.94	5.10
76%–100%	1.73	0.91	3.30	1.68	0.85	3.32	2.09	0.91	4.78
STRUCTURAL ASPECTS									
**Frequency of contact **		<0.001			0.178			0.733	
0%–25%	1.00			1.00			1.00		
26%–50%	1.07	0.64	1.79	0.99	0.56	1.74	0.91	0.47	1.76
51%–75%	1.26	0.78	2.03	1.21	0.72	2.04	0.84	0.45	1.54
76%–100%	1.84	1.19	2.83	1.39	0.87	2.24	1.01	0.57	1.79
**Network closure**		0.325			0.796			0.366	
Not all friends are friends	1.00			1.00			1.00			
All friends are friends	0.90	0.73	1.11	1.03	0.82	1.30	1.13	0.87	1.48
**Friends live in the same neighborhood**		0.220			0.505			0.824	
0%–25%	1.00			1.00			1.00		
26%–50%	1.00	0.77	1.29	0.98	0.74	1.30	1.00	0.73	1.39
51%–75%	1.20	0.89	1.62	1.25	0.89	1.74	1.19	0.80	1.76
76%–100%	1.31	0.98	1.75	1.15	0.83	1.59	0.96	0.65	1.43

n = 2,942; Model 1: Adjusted for gender and parents’ country of birth; Model 2: Gender, parents’ country of birth, parents’ social class, civil status, school grades, ego’s alcohol consumption, ego’s physical activity, and ego’s eating habits; Model 3: Gender, parents’ country of birth, parents’ social class, civil status, school grades, ego’s alcohol consumption, ego’s physical activity, ego’s eating habits and other network characteristics.

**Table 4:** Interaction between friends who smoke and other network characteristics and sociodemographic variables, 19-year-old men and women (adjusted for parents’ country of birth, social class, civil status, school grades, gender, ego’s alcohol consumption, ego’s physical activity and ego’s eating habits), prevalence ratios (PR).

	PR	95% CI/*p*-value	
FRIENDS’ HEALTH BEHAVIORS			
Friends smoke*friends physically active		<0.001	
Few smoke*few active	1.00		
Few smoke*many active	0.66	0.46	0.94
Many smoke*few active	7.14	5.16	9.87
Many smoke*many active	4.55	3.15	6.57
Friends smoke*friends eat healthy food		<0.001	
Few smoke*few eat healthy	1.00		
Few smoke*many eat healthy	0.58	0.40	0.84
Many smoke*few eat healthy	5.91	4.40	7.94
Many smoke*many eat health	6.21	4.36	8.86
RELATIONSHIP CONTENT			
Friends smoke*relationship quality		<0.001	
Few smoke*low quality	1.00		
Few smoke*high quality	1.33	0.71	2.49
Many smoke*low quality	3.85	1.69	8.75
Many smoke*high quality	10.60	5.65	19.88
Friends smoke*trust		<0.001	
Few smoke*low trust	1.00		
Few smoke*high trust	0.92	0.54	1.57
Many smoke*low trust	3.46	1.72	6.97
Many smoke*high trust	7.58	4.42	12.97
Friends smoke*discuss problems		<0.001	
Few smoke*few to discuss	1.00		
Few smoke*many to discuss	1.09	0.66	1.80
Many smoke*few to discuss	6.07	3.14	11.74
Many smoke*many to discuss	8.29	5.03	13.64
STRUCTURAL ASPECTS			
Friends smoke*frequency of contact		<0.001	
Few smoke*low frequency	1.00		
Few smoke*high frequency	0.96	0.63	1.46
Many smoke*low frequency	5.20	2.87	9.40
Many smoke*high frequency	7.53	4.97	11.42
Friends smoke*closure		<0.001	
Few smoke*not all friends are friends	1.00		
Few smoke*all friends are friends	1.04	0.73	1.49
Many smoke*not all friends are friends	7.29	5.07	10.47
Many smoke*all friends are friends	7.94	5.56	11.33
Friends smoke*friends in the same neighborhood		<0.001	
Few smoke*few live in neighborhood	1.00		1.00
Few smoke*many live in neighborhood	0.89	0.60	1.33
Many smoke*few live in neighborhood	6.47	4.88	8.59
Many smoke*many live in neighborhood	9.15	6.37	13.16
SOCIODEMOGRAPHIC VARIABLES			
Friends smoke*social class		<0.001	
Few smoke*non-manual	1.00		
Few smoke*manual	1.10	0.78	1.56
Many smoke*non-manual	8.06	5.83	11.15
Many smoke*manual	7.50	5.26	10.69
Friends smoke*parents country of birth		<0.001	
Few smoke*Swedish born	1.00		
Few smoke*foreign born	0.63	0.44	0.90
Many smoke*Swedish born	7.61	5.56	10.42
Many smoke*foreign born	4.55	3.23	6.43
Friends smoke*gender		<0.001	
Few smoke*man	1.00		
Few smoke*woman	2.04	1.44	2.91
Many smoke*man	5.84	4.09	8.35
Many smoke*woman	18.24	12.89	25.81

n = 2,942.

Having a high percentage of friends who are physically active is inversely associated with the outcome, meaning that having physically active friends reduces the risk for smoking (Model 3). More specifically, the risk of daily smoking is 0.65 (CI 0.42. 1.00) if 76%–100% of the network members are physically active after adjustment for control variables and other network characteristics (Model 3). The results suggest no significant association between other network characteristics (indicators of relationship content and structural aspects) and daily smoking after adjustment for control variables.

Having a large percentage of smokers in one’s network was the most important risk factor for daily smoking according to the results in Table 3. In the final table we therefore present results on whether the association between peer smoking and daily smoking is modulated by other network characteristics and sociodemographic factors. The findings in Table 4 suggest that egos who know many smokers who at the same time are physically inactive are at higher risk for daily smoking (PR 7.14 CI 5.16. 9.87) when compared to the reference group (few smoke and few physically active) but also when compared to those with smoking peers who are physically active (PR 4.55 CI 3.15. 6.57). Furthermore, those who have social contacts of high quality and many smoking peers (PR 10.60 CI 5.65. 19.88) are at higher risk for daily smoking than the reference group (few smoke and low relationship quality) but also when compared to those with low relationship quality and a high percentage of smokers in their network (PR 3.85 CI 1.69. 8.75). Moreover, high trust in peers increases the risk for daily smoking if those peers smoke (PR 7.58 CI 4.42. 12.97) while the risk of smoking is lower among individuals with low trust in smoking friends (PR 3.46 CI 1.72. 6.97). Finally, the propensity to discuss problems with peers increases the risk for smoking when these friends smoke (PR 8.29 CI 5.03. 13.64). Moreover, the results in Table 4 suggest that having many smoking peers who live in the same neighborhood increases the risk for daily smoking (PR 9.15 CI 6.37. 13.16), while the risk is lower if friends smoke and do not live in the same neighborhood (PR 6.47 CI 4.88. 8.59).

The results in Table 4 also reveal that women with many peers who smoke are at much higher risk for daily smoking (PR 18.24 CI 12.89. 25.81) compared to men who know many smokers (PR 5.84 CI 4.09. 8.35). The results further suggest that egos with many smoking friends of a non-manual class background are at somewhat higher risk for daily smoking (PR 8.06 CI 5.83. 11.15). Individuals with Swedish-born parents who know many smokers are also somewhat more at risk for daily smoking (PR 7.61 CI 5.56. 10.42) when compared to individuals with foreign-born parents who know many smokers (PR 4.55 CI 3.23. 6.43). This finding may indicate that people with foreign-born parents are less influenced by their peers.

## 4. Discussion

This study examined the influence of network characteristics (friends’ health behaviors, relationship content, and structural aspects) on the risk of smoking among Swedish young adults at the age of 19. We especially examined interactions between friends’ smoking behavior and other network characteristics on the risk of smoking. First of all, the results suggest that having a large percentage of smokers in one’s social network is by far the most important risk factor for daily smoking. This finding is in line with numerous other studies [2,9,10,11,12,13]. Having peers who smoke can lead adolescents and young adults to smoke because they see role models who smoke in their environment, view smoking favorably, and experience fewer punishments and more rewards as a consequence of smoking [9]. The findings also suggest that having many physically active friends reduces the risk for smoking, even after adjusting for sociodemographic variables and other network characteristics. This may indicate that other adverse health behaviors among peers may occasionally serve as behavioral influences on the decision to smoke [9]. Accordingly, it has been suggested that social networks may influence health related norms that, in turn, affect behaviors such as alcohol and cigarette consumption and physical activity [16,25]. Healthier norms and behaviors among members of the network in general may contribute to better and more positive health behaviors among egos and consequently decrease the risk of smoking, while networks dominated by individuals who engage in risky health behaviors may contribute to adverse behaviors [6,8,26,27,28]. We found no significant main associations between other network characteristics and the risk for daily smoking after adjusting for possible confounders. Accordingly, network characteristics related to relationship content, such as relationship quality, trust and propensity to discuss problems with friends, were not independently associated with daily smoking. Furthermore, we did not find any significant associations between structural aspects of social networks, such as frequency of contact, network closure or percentage of friends living in the same neighborhood, and the risk of daily smoking.

Having a large percentage of smokers in the network was the most important risk factor for daily smoking. Through interaction analysis we examined whether the association between peer smoking and daily smoking was modulated by other network characteristics and sociodemographic factors. The findings suggest particularly strong interactions between the percentage of smokers in the network and aspects of relationship content and the risk for smoking. High quality, high trust, and the propensity to discuss problems with friends increased the risk for daily smoking when peers smoke. Although most previous studies have stressed the positive aspects of relationship content and social support for health and health behaviors [16], our findings suggest that such network features may in fact increase the risk for smoking in combination with peer smoking. This is consistent with some previous evidence [18,21,22,23]. It may be that supportive friendships result in more opportunities for influence processes leading to similarities in smoking behavior among friends [19,20]. These findings also emphasize the downsides of social networks and social support as they may, in some instances, influence health behaviors negatively. Accordingly, some studies have emphasized these downsides when such networks are dominated by risky behaviors [6,26,27,28].

Some of the structural aspects of social networks such as a large percentage of peers living in the same neighborhood and meeting friends more often increased the risk for smoking when peers smoke. These findings suggest that peer influence on smoking behavior is stronger when friends live in close proximity and when friends meet more often. It is possible that such network characteristics lead to an additionally increased risk of daily smoking when friend’s attitudes, norms and behaviors support smoking because of their own smoking behavior. Finally, the findings suggest strong associations between peer smoking and gender. Women with a high percentage of smokers in their networks were at much higher risk for daily smoking than men with many smoking friends. Hence, women seem to be much more influenced by their peers’ smoking status than are men. Accordingly, it has been argued that women are subject to greater social pressure [35] and are more susceptible to social influences [36]. Nonetheless, this finding contradicts some earlier network studies that suggested that men’s smoking behavior is more socially influenced by their peers [37,38].

The data used in the present study are unique in that they contain detailed information on friendship networks in a cohort of young adults. There are some limitations and weaknesses in the data. The use of a name generator that limited the number of friends to a maximum of five may have limited these individuals’ ability to name all of their friends and their potential influence on smoking behavior. Another issue concerns the use of self-reported measures of smoking and network characteristics. Social desirability bias maintains that respondents tend to represent themselves in a favorable light [39]. which may lead to an underestimation of smoking rates. Another related weakness of the study is the fact that information on alters was given by egos. It could be argued that it is not the alter’s actual behavior that matters in terms of risk for daily smoking but rather ego’s perception of the alter’s behavior. It should also be mentioned that the present study was based on a stratified sample in terms of ethnicity. Although we adjusted for the parents’ country of birth in our empirical analyses, the stratified sampling procedure may, to some extent, limit our ability to generalize the findings to the entire Swedish population. Nevertheless, additional analyses of each separate group based on parents’ country of birth suggested no difference between groups in the association between network characteristics and daily smoking (not shown). Furthermore, the response rate was fairly low (51.6%) in the survey used. It may be that a larger number of smokers were included in the non-response. Finally, the most important limitation concerns causality. Since the present study was based on cross-sectional data, it was not possible to discern empirically whether network characteristics *per se* had a causal effect on smoking. It might be that young adults select friends who have the same smoking status as themselves, in which case the association between peer smoking and ego smoking would reflect homophily. It might also be that many of the interaction effects are due to selection processes. For instance, smokers may tend to form relationships of higher quality with other smokers. Future studies should consider the use of alternative methods such as path analysis or structural equation modeling using longitudinal data.

## 5. Conclusions

To conclude, this study suggests that social network analysis can contribute to our knowledge of smoking behavior among young adults. In line with previous findings, the percentage of smokers seems to be the most important network characteristic for smoking behavior. However, this study also underlines the importance of considering the interactions between peer smoking and other network characteristics, such as relationship content (relationship quality, trust and propensity to discuss problems), friends’ other health behaviors (physical activity and eating habits) and structural aspects of the network (frequency of contact, network closure and share of friends in the neighborhood). It seems especially important to acknowledge that traditionally positive aspects of social networks may occasionally provide more opportunities for influence processes that lead to smoking. Women were also much more socially influenced by peer smoking than men. From a policy perspective, this may be important to acknowledge. This is especially relevant when considering the increasing smoking rates among adolescent girls [1]. Nevertheless, in order to draw conclusions about whether our findings reflect peer selection or peer influence longitudinal data is suggested.
